# Single-Cell Sequencing of a Bile Sample From an Acute Cholecystitis Patient

**DOI:** 10.7759/cureus.98748

**Published:** 2025-12-08

**Authors:** Mari Tohya, Kazunori Murase, Masaaki Minagawa, Akio Saiura, Ichiro Nakagawa, Teruo Kirikae, Shin Watanabe

**Affiliations:** 1 Division of Biomedical Food Research, National Institute of Health Sciences, Kanagawa, JPN; 2 Department of Microbiology, Graduate School of Medicine, Kyoto University, Kyoto, JPN; 3 Department of Hepatobiliary-Pancreatic Surgery, Graduate School of Medicine, Juntendo University, Tokyo, JPN; 4 Department of Hepatobiliary‐Pancreatic Surgery, Graduate School of Medicine, Juntendo University, Tokyo, JPN; 5 Department of Microbiome Research, Graduate School of Medicine, Juntendo University, Tokyo, JPN; 6 Department of Emergency Medicine, Graduate School of Medicine, Juntendo University, Tokyo, JPN

**Keywords:** acute cholecystitis, e coli: escherichia coli, shigella, single-cell sequencing, whole-genome sequencing

## Abstract

Single-cell sequencing is a novel approach to genome sequencing of clinical samples. However, there are only few studies using single-cell sequencing of genomes for bacterial infections. A 71-year-old woman presented to the emergency department with epigastric pain, 38.5°C fever, and a history of hypertension and hyperuricemia. From blood test results, acute cholecystitis was suspected. The surgery went well and bilirubin calcium stones were found in the gallbladder. Single-cell sequencing was used to investigate a bile sample from a patient with acute cholecystitis. The sample, cultured on a MacConkey agar plate, produced four colonies, all identified as *Escherichia coli* by bacteriological and biochemical properties. Whole genome sequences of the four strains were determined using the single-cell amplified genome (SAG) sequencing technique. The average nucleotide identity (ANI) and digital DNA-DNA hybridization (dDDH) values of all four were 99.98-100% and 100%, respectively, indicating that they were the same bacterial species. Compared with type strains, these four strains were closest to *Shigella sonnei *(ANI 98.65-98.66%; dDDH 88.5%) than *E. coli* (ANI 96.79-96.80%; dDDH 74.2%), despite lacking *stx1*, *stx2* and *ipaH, *which *Shigella* species harbor. 16S metagenome analysis identified *E. coli* as the predominant bacterial genome in the sample, comprising 93.15%. SAG raw data had a relatively high level of quality, with 98.4-98.7% of the read numbers used after quality trimming. However, the genome sequencing coverage was only 9.45-42.88% when compared to a complete genome of an isolate with a mapping quality set above 99%, resulting in gaps compared to conventional whole genome sequence data of these isolates. The procedures of the SAG sequencing technique should be revised to improve the sequencing coverage and reduce gaps in the sequence data. Nonetheless, single-cell genome sequencing can provide novel information for bacterial infections.

## Introduction

Single-cell sequencing is an underutilized approach to genome sequencing of clinical samples from patients suspected of infections, as well as microbiota samples from humans and animals [1-3]. Single-cell sequencing may recover higher species richness in microbiota compared with culture-based approaches [4], i.e., it allows one to investigate bacterial diversity and heterogeneity, including taxonomic distribution and strain diversity within a sample [1,5]. Regarding bacterial infections, there have been few studies using single-cell sequencing of genomes, because the quality of the obtained sequences has not been well evaluated [1].

In this study, we sequenced the whole genome of single-cell bacteria from a bile sample taken from a patient with acute cholecystitis. We compared these sequences to those of bacterial isolates from the same sample to assess the quality and structure of the single-cell sequencing data and to evaluate the potential clinical significance of this technique. Single-cell sequencing has not yet been applied in routine clinical diagnostic settings. However, with the rapid advancement of genome sequencing technologies, it is expected that this approach will eventually become feasible for clinical use. By directly comparing single-cell and isolate-derived genomes, we assessed the reliability of single-cell sequencing and examined its potential clinical significance, particularly in cases where conventional culture-based methods may be insufficient. Although single-cell amplified genome (SAG) sequencing has shown utility in analyzing uncultured bacterial populations, its applicability to biliary specimens remains largely unexplored. Bile samples often contain low microbial biomass and may pose technical challenges for single-cell genome recovery. Therefore, evaluating the feasibility of SAG for bile analysis is an essential first step toward establishing its potential future use in the clinical investigation of biliary infections. The case and limitations of current procedures of single-cell sequencing are discussed.

Case details

A 71-year-old woman presented to the emergency department of Juntendo University Hospital in March 2019 with epigastric pain, 38.5°C fever, and a history of hypertension. Four days later, she developed severe right hypochondoralgia. Initial laboratory examinations indicated a white blood cell (WBC) count of 8,600/mm^3^, a C-reactive protein (CRP) concentration of 146.8 mg/l, and a total serum bilirubin level of 0.92 mg/dL. Based on these clinical findings and blood test results, acute cholecystitis was suspected. Computed tomography revealed a stone in the neck of the gallbladder, which confirmed our diagnosis of acute cholecystitis. Ahead of emergency surgery, the patient was given 1 g of cefmetazole. The surgery went well, but adhesions caused by inflammation of the gallbladder required laparotomy. Bilirubin calcium stones were found in the gallbladder. The pathological findings showed severe acute cholecystitis, but no malignancy.

## Materials and methods

Sample collection and bacterial identification

During a cholecystectomy procedure, a bile sample was collected from the gallbladder of a patient suspected of acute cholecystitis. The collection was performed by aspirating the sample using an 18-gauge needle. The bile sample was collected in a sterilized Spitz tube and then transported to the clinical laboratory in an anaerobic porter to maintain its integrity. Additionally, any remaining sample that was not needed for immediate testing was promptly frozen at a temperature of -80℃ for further analysis. The sample was inoculated on MacConkey agar and cultured in an aerobic chamber at 37℃ for 24 h. Eight colonies grown on the agar were isolated to identify the bacterial species and stored at -80℃. Bacterial identification was conducted using MALDI Biotyper (Bruker, Billerica, MA).

Drug susceptibility tests

Drug susceptibility tests by the broth microdilution method followed the guidelines of the Clinical Laboratory Standards Institute (CLSI M100-S29). Minimum inhibitory concentrations (MICs) of arbekacin (ABK), amikacin (AMK), ceftazidime (CAZ), imipenem (IPM), meropenem (MPM), ciprofloxacin (CIP), and colistin (CST) were determined.

Whole genome sequencing

All eight colonies displayed similar morphology suggestive of the same bacterial species; therefore, four colonies were randomly selected to avoid selection bias. Whole-genome sequencing of the four strains was performed using two sequencers, MiSeq and MinION, following the manufacturers’ instructions. MiSeq sequence reads were quality trimmed and filtered using CLC Genomics Workbench v11 (CLC bio, Aarhus, Denmark), while MinION data were base called and adapter trimmed with Guppy v3.6.1 (Oxford Nanopore Technologies, Oxford, UK). The sequence data generated from both Miseq and MinION were assembled using Unicycler v0.4.6 [6]. Prokka [7] was used to annotate the assembled sequences with the assembled sequence of JU501 serving as a reference. The genetic relationship between obtained genomic data in this study was estimated by average nucleotide identity (ANI) [8] using the OrthoANIu algorithm [9] and digital DNA-DNA hybridization (dDDH) using the genome-to-genome distance calculator version 3.0 [10].

Metagenome sequencing

DNA was extracted from the bile sample with DNeasy PowerSoil Kits (QIAGEN, Hilden, Germany). A DNA library was prepared in accordance with Illumina 16S Metagenomic Sequencing Library Preparation Guide, targeting the V3-V4 region of the 16S rRNA gene. The fastq files were analyzed using EzBioCloud 16S rRNA gene-based microbiome taxonomic profiling [11].

Single-cell sequencing

SAG sequencing was performed as described in [12]. Individual bacterial cells were randomly captured in picoliter-sized gel beads and processed by in-gel lysis to extract DNA and perform whole-genome amplification. SAG libraries were prepared using the QIAseq FX DNA Library Kit (QIAGEN). Ligation adaptors were modified using TruSeq-Compatible Full-length Adapters UDI (Integrated DNA Technologies, Coralville, IA). Each SAG library was sequenced using the Illumina HiSeq 2 × 150 bp configuration.

Analysis of single-cell sequencing data

The short-sequence reads of SAG libraries and isolates were trimmed using fastp [13] and then mapped to the reference sequence using BWA-MEM [14,15]. Genomic synteny among the short-sequence reads and the reference sequences was visualized using GenomeMatcher [16]. Nucleotide mutations were detected using the Basic Variant Detection Tool in the CLC Genomics Workbench v11 with the following parameters: coverage = 10, minimum count = 10 and minimum frequency (%) = 50 [17]. Variant calling parameters were determined based on the sequencing depth obtained from the SAG datasets. The SAG with the lowest sequencing yield (SAG2; 57 MB of forward FASTQ reads) was used as the reference point, and stringent thresholds were applied to ensure reliable variant detection across all SAGs. Detection of antimicrobial resistance genes was performed using ResFinder version 4.7.2 [18,19] while virulence-associated genes were identified using VirulenceFinder version 2.0 [20,21]. For each SAG and isolate genome, the presence or absence of target genes was recorded, and results were compared to assess consistency between single-cell and isolate sequences.

## Results

Eight similar-looking, pink-colored colonies grew on MacConkey agar when the bile sample was inoculated, among which four strains were isolated. All four strains, JU501-504, were Gram-negative rods and motile. Biochemical properties of the strains determined by API 20 were identical to each other (Table 1), and API 20 reported that the strains were *Escherichia coli*. In brief, they were indole positive; β-galactosidase positive; fermentation of lactose, sucrose, and mannitol; ornithine dihydrolase positive and arginine dihydrolase negative (Table 1). MALDI-TOF MS (MALDI-Biotyper) analysis determined all four strains were* E. coli* (data not shown). They showed essentially the same drug susceptibility profiles with the following MICs: 1 µg/ml for ABK, 2µg/ml or 4 µg/ml for AMK, 0.5 µg/ml for CAZ, 0.5 µg/ml for IPM, 0.5 µg/ml for MPM, 0.5 µg/ml for CIP, and 0.5 µg/ml for CST.

**Table 1:** Phenotypic characteristics of isolates and a type strain of E. coli, NBRC 102203T, using API 20. All data were obtained in this study. +, Positive; -, Negative.

Tests	JU501	JU502	JU503	JU504	NBRC 102203^T^
Ortho-Nitro-Phenyle-Galactosidase (ONPG)	+	+	+	+	+
Arginine (ADH)	-	-	-	-	-
Lysine (LDC)	+	+	+	+	+
Ornithine (ODC)	+	+	+	+	+
Sodium citrate (CIT)	-	-	-	-	-
Sodium thiosulfate (H_2_S)	-	-	-	-	-
Urease (URE)	-	-	-	-	-
Tryptophan (TDA)	-	-	-	-	-
Indol (IND)	+	+	+	+	+
Voges-Proskauer (VP)	-	-	-	-	-
Gelatinase (GE)	-	-	-	-	-
Glucose (GLU)	+	+	+	+	+
Mannitol (MAN)	+	+	+	+	+
Inositol (INO)	-	-	-	-	-
Sorbitol (SOR)	+	+	+	+	+
Rhamnose (RHA)	+	+	+	+	+
Sucrose (SAC)	+	+	+	+	-
Melibiose (MEL)	+	+	+	+	+
Amygdalin (AMY)	-	-	-	-	-
Arabinose (ARA)	+	+	+	+	+

When whole genome sequences of JU501-504 strains isolated from the bile were determined and compared with each other, ANI and dDDH values were 99.98-100% and 100%, respectively (Tables 2, 3), indicating that all four strains belonged to the same bacterial species. Compared with the type strains, the four strains were closest to *Shigella sonnei *(ANI 98.65-98.66%; dDDH 88.5%), then *Shigella boydii* (ANI 98.55-98.63%; dDDH 88.5-88.7%), followed by *Shigella flexineri* (ANI 98.33-98.39%; dDDH 86.4%), *Shigella dysenteriae* (ANI 97.83-97.86%; dDDH 82.2%), and *E. coli* (ANI 96.79-96.80%; dDDH 74.2%). None of these strains harbored *stx1*, *stx2,* or *ipaH*, which *Shigella* species harbor. We concluded that the causative agent of acute cholecystitis in this case was drug-susceptible *E. coli*.

**Table 2:** ANI values among SAGs and type strains. ANI values were calculated among SAGs and type strains with the OrthoANIu algorithm.  ANI values are expressed as percentages, and the cut-off values of >95% indicate that they belong to the same species. ANI: average nucleotide identity, SAG: single-cell amplified genome, *S. sonnei*: *Shigella sonnei*, *S. boydii*: *Shigella boydii*, *S. flexineri*: *Shigella flexineri*, *S. dysenteriae*: *Shigella dysenteriae*, *E. coli*: *Escherichia coli. *

	SAG1	SAG2	SAG3	SAG4	JU501	JU502	JU503	JU504	S. sonnei	S. boydii	S. flexineri	S. dysenteriae	E. coli
SAG1	-	99.19	99.53	99.45	99.87	99.80	99.87	99.86	98.33	98.14	97.97	97.62	96.65
SAG2	99.19	-	99.15	99.46	99.87	99.79	99.87	99.86	98.07	97.95	97.73	97.58	96.71
SAG3	99.53	99.15	-	99.68	99.88	99.88	99.88	99.88	98.24	98.26	98.01	97.68	96.51
SAG4	99.45	99.46	99.68	-	99.86	99.87	99.79	99.87	98.40	98.49	98.15	97.69	96.66
JU501	99.87	99.87	99.88	99.86	-	100.0	99.98	100.0	98.65	98.55	98.33	97.83	96.79
JU502	99.80	99.79	99.88	99.87	100.0	-	99.98	100.0	98.65	98.55	98.33	97.83	96.79
JU503	99.87	99.87	99.88	99.79	99.98	99.98	-	99.98	98.66	98.63	98.39	97.86	96.80
JU504	99.86	99.86	99.88	99.87	100.0	100.0	99.98	-	98.65	98.55	98.33	97.83	96.79
S. sonnei	98.33	98.07	98.24	98.40	98.65	98.65	98.66	98.65	-	98.62	98.40	97.80	96.89
S. boydii	98.14	97.95	98.26	98.49	98.55	98.55	98.63	98.55	98.62	-	98.23	97.71	96.84
S. flexineri	97.97	97.73	98.01	98.15	98.33	98.33	98.39	98.33	98.40	98.23	-	97.75	96.99
S. dysenteriae	97.62	97.58	97.68	97.69	97.83	97.83	97.86	97.83	97.80	97.71	97.75	-	96.98
E. coli	96.65	96.71	96.51	96.66	96.79	96.79	96.80	96.79	96.89	96.84	96.99	96.98	-

**Table 3:** dDDH values among SAGs and type strains. dDDH values were calculated among SAGs and type strains with the genome-to-genome distance calculator version 3.0. dDDH values are expressed as percentages, and the cut-off values of >70% indicate that they belong to the same species. dDDH: digital DNA-DNA hybridization, SAG: single-cell amplified genome, *S. sonnei*: *Shigella sonnei,* *S. boydii*: *Shigella boydii*,* S. flexineri*: *Shigella flexineri*, *S. dysenteriae*: *Shigella* *dysenteriae*, *E. coli*: *Escherichia coli*.

	SAG1	SAG2	SAG3	SAG4	JU501	JU502	JU503	JU504	S. sonnei	S. boydii	S. flexineri	S. dysenteriae	E. coli
SAG1	-	94.8	96.8	96.8	98.1	98.1	98.2	98.1	86.0	86.4	84.0	80.3	74.0
SAG2	94.8	-	95.2	95.4	96.4	96.4	96.4	96.4	84.6	85.2	82.4	79.7	73.1
SAG3	96.8	95.2	-	96.4	98.3	97.9	98.3	98.3	86.4	86.4	84.4	80.5	73.5
SAG4	96.8	95.4	96.4	-	98.1	98.1	98.1	98.1	86.7	86.9	84.6	80.8	73.5
JU501	98.1	96.4	98.3		-	100.0	100.0	100.0	88.5	88.6	86.4	82.2	74.2
JU502	98.1	96.4	97.9		100.0	-	100.0	100.0	88.5	88.5	86.4	82.2	74.2
JU503	98.2	96.4	98.3		100.0	100.0	-	100.0	88.5	88.5	86.4	82.2	74.2
JU504	98.1	96.4	98.3		100.0	100.0	100.0	-	88.5	88.5	86.4	82.2	74.2
S. sonnei	86.0	84.6	86.4		88.5	88.5	88.5	88.5	-	88.7	86.8	92.6	74.6
S. boydii	86.4	85.2	86.4		88.6	88.5	88.5	88.5	88.7	-	86.7	81.1	74.7
S. flexineri	84.0	82.4	84.4		86.4	86.4	86.4	86.4	86.8	86.7	-	79.5	74.4
S. dysenteriae	80.3	79.7	80.5		82.2	82.2	82.2	82.2	82.6	81.1	79.5	-	73.5
E. coli	74.0	73.1	73.5		74.2	74.2	74.2	74.2	74.6	74.7	74.4	73.5	-

ANI values shown in Table 2 were 99.15-99.86% among SAGs and 99.79-99.88% when SAGs were compared with isolates. When compared with those of type strains, SAG1 and SAG2 were mostly similar to the type strain of *S. sonnei*, but SAG3 and SAG4 were mostly similar to the type strain of *S. boydii*. dDDH values shown in Table 3 were 95.2-96.8% among SAGs and 96.4-98.3% when SAGs were compared with isolates. dDDH analysis also showed that SAG1 and SAG2 were mostly similar to the type strain of* S. sonnei*, but SAG3 and SAG4 were mostly similar to the type strain of *S. boydii*. These bacterial identification results for SAG3 and SAG4 were different from those of the isolates which were mostly similar to *S. sonnei*.

**Figure 1:**
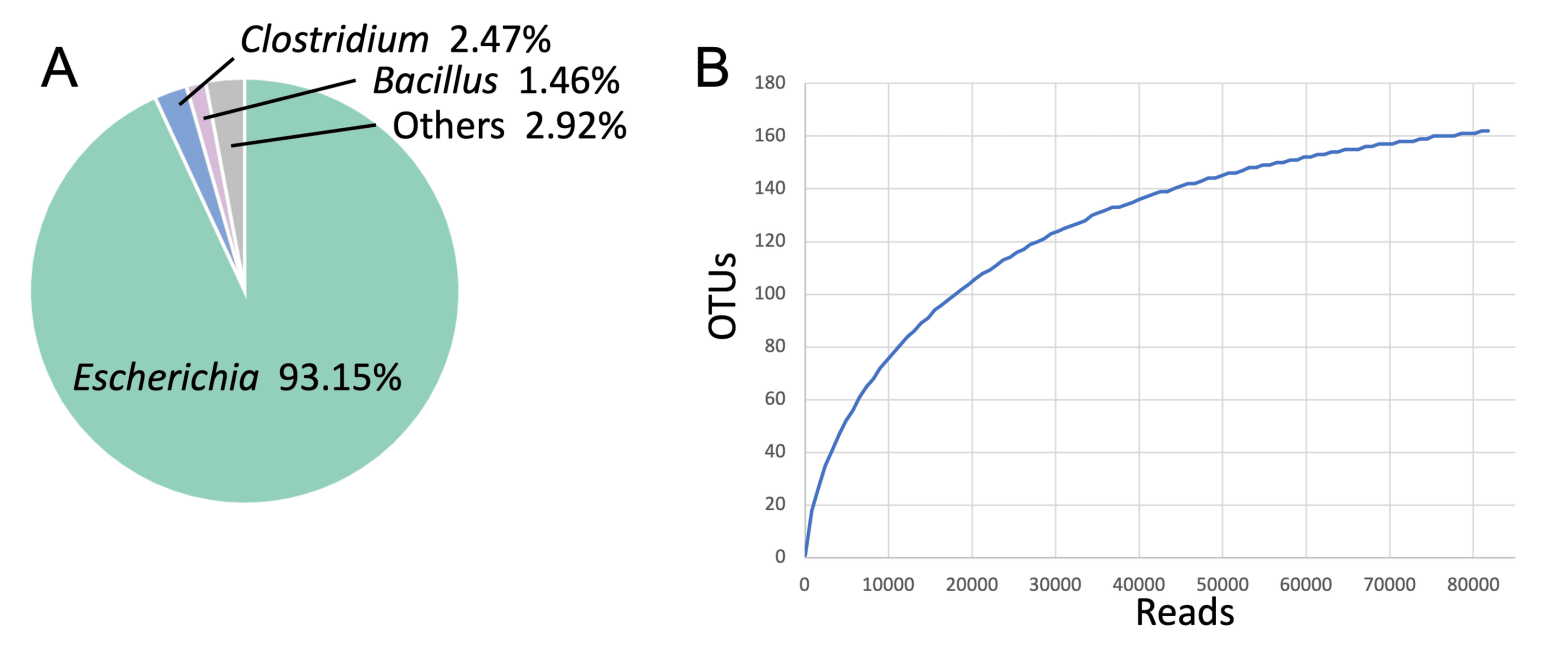
Microbiome compositions and rarefaction curves of operational taxonomic units (OTUs). A) Microbiome composition at the genus level. B) Rarefaction curves of OTUs.

The 16S metagenome analysis revealed that most of the bacterial genomes were from *E. coli* (93.15%), followed by genus *Clostridium* (2.47%) and *Bacillus* (1.46%) (Figure 1A). These results suggest that *E. coli* may be the causative agent of acute cholecystitis in this case. The rarefaction curve of operational taxonomic units exhibited a plateau (Figure 1B). The α-diversity indices, including OTU, Chao1, and ACE, were calculated as162, 177.0, and 177.8, respectively.

Four SAGs, SAG1 to SAG4, were obtained from the bile sample. Table 4 shows that their sequence data had a high level of quality with 98.4-98.7% of the read numbers of SAG raw data and 72.2-76.3% of those of the isolates used as trimmed data. Table 5 shows that the reference sequence coverage of SAGs was lower than that of conventional whole genome sequence data of strains, with coverages of 18.06-54.61%, 9.45-42.88%, and 4.68-32.68% when the mapping quality was set at >90%, >90%, and > 99.9%, respectively, whereas coverage of the genomes was >99.8% regardless of mapping quality when whole genome sequences were determined using the strains.

**Table 4:** Coverage of consensus sequences of SAGs and isolates compared with those of reference sequences (JU501). SAG: single-cell amplified genome

		SAG1	SAG2	SAG3	SAG4	JU501	JU502	JU503	JU504
Mapping quality >90%	Consensus seq (bp)	2,346,045	862,044	2,575,843	2,606,122	4,772,063	4,772,063	4,764,637	4,772,063
	Coverage (%)	49.16	18.06	53.98	54.61	100	100	99.84	100
Mapping quality >99%	Consensus seq (bp)	1,768,906	450,941	2,022,488	2,046,062	4,772,063	4,772,063	4,764,637	4,772,063
	Coverage (%)	37.07	9.45	42.38	42.88	100	100	99.84	100
Mapping quality>99.9%	Consensus seq (bp)	1,238,272	223,398	1,532,174	1,559,729	4,772,063	4,772,063	4,764,637	4,772,063
	Coverage (%)	25.95	4.68	32.11	32.68	100	100	99.84	100

**Table 5:** Quality of raw sequence data of SAGs and isolates. SAG: single-cell amplified genome

Sample		Raw data		Trimmed data (q>10, length> 30bp)	
		No. of pair-end reads	No. of bases	No. of pair-end reads (% of remaining reads)	No. of bases (% of remaining bases)
Sequencing data of single-cell	SAG1	663,778	100,230,478	653,206 (98.4)	91,621,210 (91.4)
	SAG2	308,146	46,530,046	303,568 (98.5)	42,098,574 (90.5)
	SAG3	842,092	127,155,892	830,006 (98.6)	117,379,526 (92.3)
	SAG4	736,018	111,138,718	726,346 (98.7)	99,992,646 (90.0)
Sequencing data of isolates	JU501	1,122,964	306,436,166	843,734 (75.1)	222,494,164 (72.6)
	JU502	832,400	235,171,979	601,018 (72.2)	210,748,082 (89.6)
	JU503	1,040,696	285,081,080	786,210 (75.5)	208,645,403 (73.2)
	JU504	971,252	263,904,417	741,076 (76.3)	194,702,609 (73.8)

**Figure 2:**
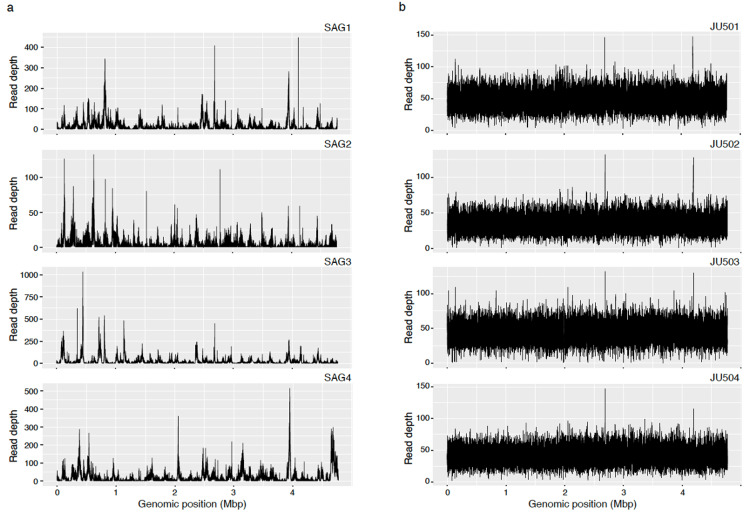
Read depth of sequencing data of SAGs and isolates. a: Read depth of SAGs’ sequencing data. b: Read depth of isolates’ sequencing data. SAG: single-cell amplified genome

**Figure 3:**
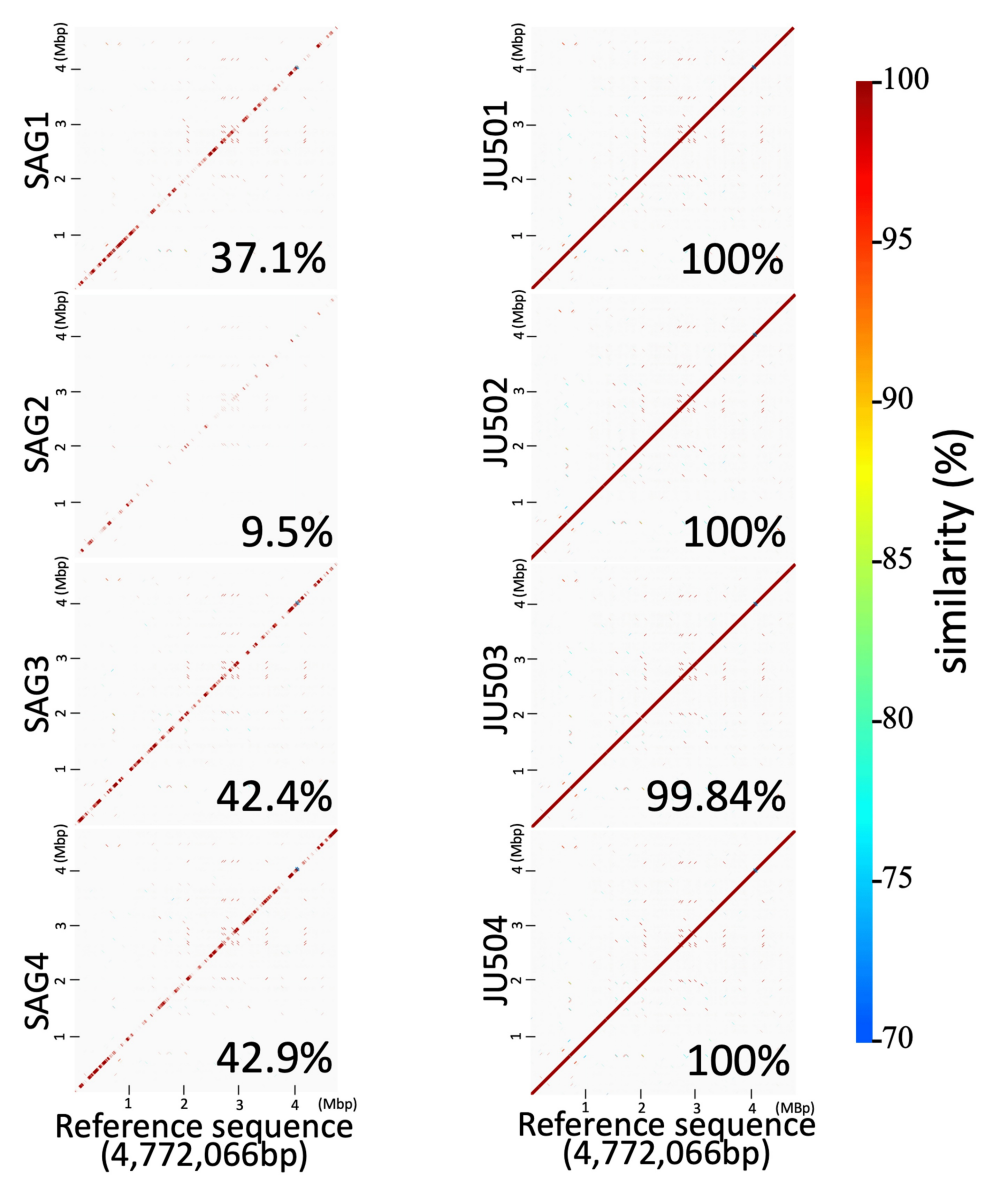
Comparison of consensus sequences with the reference sequence using GenomeMatcher. GenomeMatcher was used to compare the reference sequence (JU501) with the consensus sequences of SAGs (left column) and isolates (right column). The positions on genome of the reference sequence and sequencing data of SAGs and isolates are indicated on the x-axis and y-axis, respectively. The dot color indicates the percentage similarity, as indicated in the right vision. Mapping coverage percentages were indicated at bottom right of each figure. SAG: single-cell amplified genome

**Figure 4:**
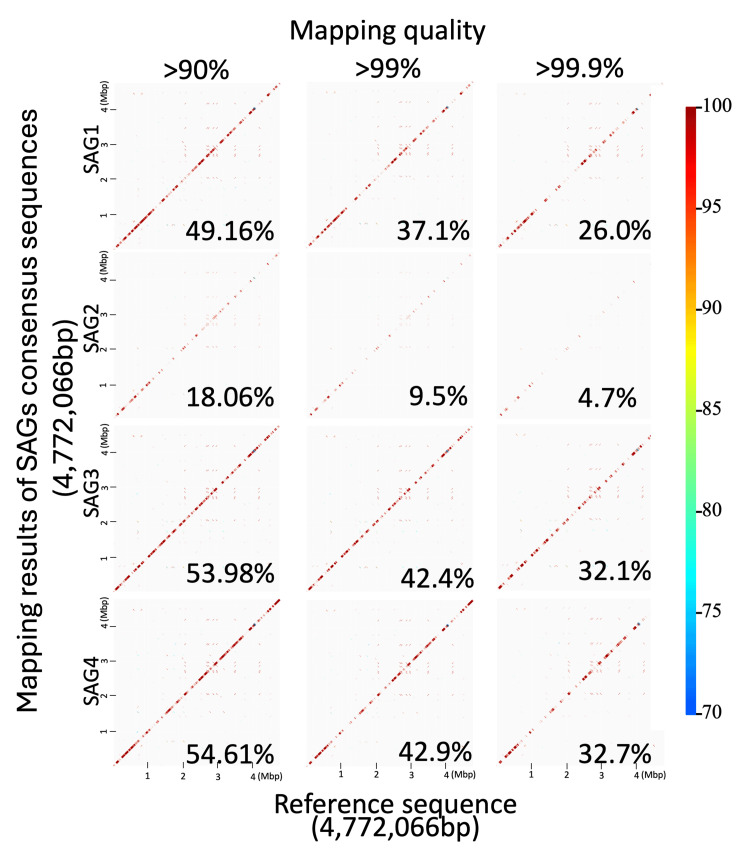
GenomeMatcher analysis of coverages of SAGs’ consensus sequences. The consensus sequences of three mapping quality condition, >90%, >99% and >99.9%, were compared using GenomeMatcher and these coverage percentages were shown at bottom right. The positions on genome of the reference sequence and sequencing data of SAGs are indicated on the x-axis and y-axis, respectively. The dot color indicates the percentage similarity, as indicated in the right vision. SAG: single-cell amplified genome

Sequencing depth data given in Table 6 and Figure 2 show that standard deviations (SDs) of SAG sequencing depth varied widely ranging from 7.2 to 48.3 compared to those of the whole genome sequences of the strains. These data suggest that sequence data of SAGs had effective quality for genome analyses, but the coverage of the reference sequence was lower than that of conventional whole genome sequence data. GenomeMatcher images show that gaps in SAG sequence data were scattered in the whole genome sequences of the reference sequence of JU501 (Figures 3, 4), indicating that gaps occurred independently of genome structures.

**Table 6:** Sequencing depth of SAGs and isolates *SD: standard deviation, SAG: single-cell amplified genome

sample		Sequencing depth	
		mean	SD*
Sequencing data of single-cell	SAG1	15.0459	25.0406
	SAG2	4.0602	7.1618
	SAG3	24.3129	48.2631
	SAG4	20.6863	33.8053
Sequencing data of isolates	JU501	46.4767	11.5577
	JU502	34.5892	9.6227
	JU503	43.5912	11.3982
	JU504	40.6687	10.6696

The nucleotide mutations between SAG sequences and the reference sequence of JU501 are shown in Table 7. Three to 31 mutations were detected in each SAG sequence. Although two mutations, at nucleotide positions 3,945,520 and 3,945,559, were detected in all four SAG sequences, the other mutations were not the same in all the sequences.

**Table 7:** Variant detection from each SAG's data. *: stop codon. SAG: single-cell amplified genome

	Reference region	Reference	Allele	Product	Amino acids change	Count	Coverage	Frequency
SAG1	2667737	A	T	rluF	Val79Asp	10	19	52.63
	2667740..2667741	AT	TA	rluF	Ile78Tyr	13	21	61.90
	2667746..2667747	AC	CG	rluF	Val76Arg	13	24	54.17
	2858483	A	G	polA	Leu377Pro	26	26	100
	3945520	T	G	Non coding region	-	60	62	96.77
	3945559	T	C	Non coding region	-	63	64	98.44
SAG2	2391142	T	C	Non coding region	-	13	13	100
	3945520	T	G	Non coding region	-	16	16	100
	3945559	T	C	Non coding region	-	15	15	100
SAG3	158217	T	G	Non coding region	-	18	18	100
	353886	A	C	LOCUS_03370: hypothetical protein	Asp53Ala	46	88	52.27
	353892	A	C	LOCUS_03370: hypothetical protein	Asn55Thr	53	87	60.91
	353895	A	T	LOCUS_03370: hypothetical protein	Tyr56Phe	52	95	54.74
	353900^353901	-	GCGC	LOCUS_03370: hypothetical protein	Glu58fs	89	123	72.36
	354603	T	AA	LOCUS_03390: membrane protein	Leu48fs	65	121	53.72
	354607..354608	CT	GC	LOCUS_03390: membrane protein	Trp50Arg	58	98	59.1836735
	354613..354614	TC	AT	LOCUS_03390: membrane protein	Tyr51*	61	86	70.9302326
	354616..354618	GGC	AAA	LOCUS_03390: membrane protein	Ala53Lys	66	91	72.5274725
	354620	G	C	LOCUS_03390: membrane protein	Ala54Pro	59	80	73.75
	354627..354628	TC	AA	LOCUS_03390: membrane protein	Val56Glu	46	67	68.6567164
	354630..354631	TC	GA	LOCUS_03390: membrane protein	Val57Gly	43	65	66.1538462
	354634..354635	TT	AC	Non coding region	-	41	57	71.9298246
	354638	T	C	Non coding region	-	40	54	74.0740741
	354641	A	C	LOCUS_03390: membrane protein	Asn61His	41	53	77.3584906
	354643..354644	CA	AT	LOCUS_03390: membrane protein	Asn61_Arg62delinsLysTrp	40	51	78.4313725
	354646	G	C	LOCUS_03390: membrane protein	Arg62Ser	33	45	73.3333333
	354648	T	A	LOCUS_03390: membrane protein	Leu63Gln	33	43	76.744186
	354651..354652	TT	GG	LOCUS_03390: membrane protein	Leu64Arg	26	37	70.2702703
	453328^453329	-	CC	LOCUS_04330: cyclopropane-fatty-acyl-phospholipid synthease	Ala315fs	52	100	52
	453335	T	A	LOCUS_04330: cyclopropane-fatty-acyl-phospholipid synthease	Asn312Ile	54	100	54
	1403510	T	C	gsiD	Ile15Met	10	14	71.4285714
	1582986	C	T	Non coding region	-	11	19	57.8947368
	1586502	A	-	Non coding region	-	30	53	56.6037736
	3151073	A	G	Non coding region	-	19	20	95
	3279714	C	T	LOCUS_29900: hypothetical protein	Gly35Glu	11	16	68.75
	3279720^3279721	-	ACGGAGCA	LOCUS_29900: hypothetical protein	Ala33fs	11	19	57.8947368
	3279727	T	C	LOCUS_29900: hypothetical protein	Met31Val	12	20	60
	3311628	T	A	nikC	His270Leu	11	11	100
	3945520	T	G	Non coding region	-	54	56	96.4285714
	3945559	T	C	Non coding region	-	44	44	100
SAG4	85544	T	C	LOCUS_00760: glycosyl transferase	Tyr90His	11	11	100
	2459971	C	T	ytfA	Trp103*	13	13	100
	3151073	A	G	Non coding region	-	50	51	98.0392157
	3312119	G	A	Non coding region	-	10	10	100
	3558765	C	T	Non coding region	-	15	17	88.2352941
	3671471	G	-	exuT	Leu84fs	22	22	100
	3938103	A	C	spaP	Tyr124Ser	90	93	96.7741935
	3945520	T	G	Non coding region	-	91	95	95.7894737
	3945559	T	C	Non coding region	-	76	82	92.6829268

Analysis of antimicrobial resistance genes showed that both SAG1-4 and JU501-504 were negative, indicating no detectable resistance determinants. In contrast, differences were observed in the profiles of virulence-associated genes between SAGs and JU501-504, with SAGs generally detecting fewer genes (Table 8). 

**Table 8:** Comparison of virulence-associated genes detected in JU501-504 and SAG1-4. The genes detected using VirulenceFinder 2.0 are summarized in the table.

Genome	Detected virulence genes
JU501	*csgA*, *fdeC*, *fimH*, *gad*, *hlyE*, *iss*, *lpfA*, *nlpl*, *ompT*, *terC*, *yehA*, *yehB*, *yehC*, *yehD*
JU502	*csgA*, *fdeC*, *fimH*, *gad*, *hlyE*, *iss*, *lpfA*, *nlpl*, *ompT*, *terC*, *yehA*, *yehB*, *yehC*, *yehD*
JU503	*csgA*, *fdeC*, *fimH*, *gad*, *hlyE*, *iss*, *lpfA*, *nlpl*, *ompT*, *terC*, *yehA*, *yehB*, *yehC*, *yehD*
JU504	*csgA*, *fdeC*, *fimH*, *gad*, *hlyE*, *iss*, *lpfA*, *nlpl*, *ompT*, *terC*, *yehA*, *yehB*, *yehC*, *yehD*
SAG1	*csgA*, *fimH*, *gad*, *hlyE*
SAG2	fimH
SAG3	*fimH*, *hlyE*, *ompT*, *terC*
SAG4	*fimH*, *gad*, *nlpl*, *ompT*, *yehA*, *yehB*, *yehC*, *yehD*

## Discussion

The limited genome coverage obtained from SAG data may affect the reliability of pathogen identification and hinder the detection of clinically relevant genes, particularly in low-biomass samples such as bile. To improve the coverage of SAG sequences (Table 5) and reduce gaps in SAG sequence data (Figure 1), some steps in the SAG sequencing process should be revised, including the conditions for cell lysis and DNA extraction using enzyme mixtures, as well as DNA amplification from a single genome in gel beads with polymerases. Zhang et al. successfully prepared megabase-size DNA from multiple organisms using an enzymatic cocktail [22], and Chijiiwa et al. adopted this cocktail to extract DNA in SAG sequencing [12]. The DNA extraction procedure may be reconsidered, and the DNA amplification conditions and polymerase could be optimized.

While whole genome sequencing analysis, including ANI and dDDH, has become standard in bacterial identification, species-specific genes and biochemical properties must be taken into account. For example, while *Shigella* and *Escherichia coli* may be considered a single species based on DNA relatedness [23], they remain classified as separate species to avoid confusion in medical microbiology [24]. There is a similar difficulty in bacterial identification in *Bacillus* species, i.e., non-toxin-producing *Bacillus cereus* strains belong to the *B. anthracis* clade [25], and several members of the *Bacillus cereus* group could not be distinguished from *B. anthracis* [26].

Though using single colonies cultured from clinical samples does not fully reflect the advantages of single-cell sequencing in clinical infection samples [27], single-cell sequencing of bacteria can provide valuable information regarding their genetic response to the environmental conditions, including DNA mutations and mRNA expressions. In vivo studies have demonstrated that bile acids can damage bacterial DNA [28], leading bacteria to develop mechanisms for responding to bile [29]. Bile acids also impact mRNA expression of *E. coli* virulence genes, which may play a crucial role in surviving host defense mechanisms [30]. When performing single-cell sequencing on bacteria under such environmental conditions, it may be necessary to increase the number of samples for analysis and consider additional experiments, such as single-cell RNA sequencing. In this study, we focused on DNA-based single-cell genome sequencing because our primary aim was pathogen identification, although single-cell RNA sequencing can provide valuable information on transcriptional activity. Furthermore, in the field of gut microbiome research, applications of single-cell sequencing have been reported, revealing the presence of unculturable bacteria through the technique [12]. There is also significant potential for detecting unculturable bacteria in infectious disease specimens. Moreover, it holds considerable promise for effectively addressing mixed infections caused by multiple bacterial species.

Comparison of virulence-associated genes between SAGs and isolate genomes revealed that SAGs generally detected fewer genes. Several genes that were present in the isolate genomes were not identified in the corresponding SAGs, likely reflecting the incomplete genome coverage inherent to single-cell sequencing.

The limitation of this study is that the results of the single-cell genome analysis and the analysis of clinical isolates compared in this study are analyses using a single case. In addition, the absence of negative controls represents a methodological limitation, as single-cell capture procedures in low-biomass samples are susceptible to environmental or reagent-derived contamination. Hence, it is difficult to conclude on the validity of the single-cell analysis in this study. With the emergence of various genome analysis methods, their application to clinical specimens may yield useful results when used to understand the genetic characteristics of bacteria.

## Conclusions

Relying solely on a clinical case study may not provide sufficient evidence to draw conclusions about the effectiveness of single-cell sequencing. Nonetheless, single-cell genome sequencing can provide novel information about the pathology of several bacterial infections, including acute infections. Therefore, it would be useful to introduce this technique into studies of such bacterial infections.

## References

[1] Woyke T, Doud DF, Schulz F (2017). The trajectory of microbial single-cell sequencing. Nat Methods.

[2] Kaster AK, Sobol MS (2020). Microbial single-cell omics: the crux of the matter. Appl Microbiol Biotechnol.

[3] Arikawa K, Ide K, Kogawa M (2021). Recovery of strain-resolved genomes from human microbiome through an integration framework of single-cell genomics and metagenomics. Microbiome.

[4] Lloréns-Rico V, Simcock JA, Huys GR, Raes J (2022). Single-cell approaches in human microbiome research. Cell.

[5] Ide K, Saeki T, Arikawa K (2022). Exploring strain diversity of dominant human skin bacterial species using single-cell genome sequencing. Front Microbiol.

[6] Wick RR, Judd LM, Gorrie CL, Holt KE (2017). Unicycler: resolving bacterial genome assemblies from short and long sequencing reads. PLoS Comput Biol.

[7] Seemann T (2014). Prokka: rapid prokaryotic genome annotation. Bioinformatics.

[8] Konstantinidis KT, Tiedje JM (2005). Genomic insights that advance the species definition for prokaryotes. Proc Natl Acad Sci U S A.

[9] Yoon SH, Ha SM, Lim J, Kwon S, Chun J (2017). A large-scale evaluation of algorithms to calculate average nucleotide identity. Antonie Van Leeuwenhoek.

[10] Meier-Kolthoff JP, Auch AF, Klenk HP, Göker M (2023). Genome-to-genome distance calculator 3.0. BMC Bioinformatics.

[11] Yoon SH, Ha SM, Kwon S, Lim J, Kim Y, Seo H, Chun J (2017). Introducing EzBioCloud: a taxonomically united database of 16S rRNA gene sequences and whole-genome assemblies. Int J Syst Evol Microbiol.

[12] Chijiiwa R, Hosokawa M, Kogawa M (2020). Single-cell genomics of uncultured bacteria reveals dietary fiber responders in the mouse gut microbiota. Microbiome.

[13] Chen S, Zhou Y, Chen Y, Gu J (2018). fastp: an ultra-fast all-in-one FASTQ preprocessor. Bioinformatics.

[14] Li H, Durbin R (2009). Fast and accurate short read alignment with Burrows-Wheeler transform. Bioinformatics.

[15] Li H, Durbin R (2010). Fast and accurate long-read alignment with Burrows-Wheeler transform. Bioinformatics.

[16] Ohtsubo Y, Ikeda-Ohtsubo W, Nagata Y, Tsuda M (2008). GenomeMatcher: a graphical user interface for DNA sequence comparison. BMC Bioinformatics.

[17] Moran NA, McLaughlin HJ, Sorek R (2009). The dynamics and time scale of ongoing genomic erosion in symbiotic bacteria. Science.

[18] Bortolaia V, Kaas RS, Ruppe E (2020). ResFinder 4.0 for predictions of phenotypes from genotypes. J Antimicrob Chemother.

[19] Camacho C, Coulouris G, Avagyan V, Ma N, Papadopoulos J, Bealer K, Madden TL (2009). BLAST+: architecture and applications. BMC Bioinformatics.

[20] Joensen KG, Scheutz F, Lund O, Hasman H, Kaas RS, Nielsen EM, Aarestrup FM (2014). Real-time whole-genome sequencing for routine typing, surveillance, and outbreak detection of verotoxigenic Escherichia coli. J Clin Microbiol.

[21] Malberg Tetzschner AM, Johnson JR, Johnston BD, Lund O, Scheutz F (2020). In silico genotyping of Escherichia coli isolates for extraintestinal virulence genes by use of whole-genome sequencing data. J Clin Microbiol.

[22] Zhang M, Zhang Y, Scheuring CF, Wu CC, Dong JJ, Zhang HB (2012). Preparation of megabase-sized DNA from a variety of organisms using the nuclei method for advanced genomics research. Nat Protoc.

[23] Pettengill EA, Pettengill JB, Binet R (2017). Corrigendum: phylogenetic analyses of Shigella and enteroinvasive Escherichia coli for the identification of molecular epidemiological markers: Whole-genome comparative analysis does not support distinct genera designation. Front Microbiol.

[24] Chattaway MA, Schaefer U, Tewolde R, Dallman TJ, Jenkins C (2017). Identification of Escherichia coli and Shigella Species from whole-genome sequences. J Clin Microbiol.

[25] Venkateswaran K, Singh NK, Checinska Sielaff A (2017). Non-toxin-producing Bacillus cereus Strains belonging to the B. anthracis clade isolated from the international space station. mSystems.

[26] Okinaka R, Pearson T, Keim P (2006). Anthrax, but not Bacillus anthracis?. PLoS Pathog.

[27] Zheng W, Zhao S, Yin Y (2022). High-throughput, single-microbe genomics with strain resolution, applied to a human gut microbiome. Science.

[28] Kandell RL, Bernstein C (1991). Bile salt/acid induction of DNA damage in bacterial and mammalian cells: implications for colon cancer. Nutr Cancer.

[29] Prouty AM, Brodsky IE, Falkow S, Gunn JS (2004). Bile-salt-mediated induction of antimicrobial and bile resistance in Salmonella typhimurium. Microbiology (Reading).

[30] Hamner S, McInnerney K, Williamson K, Franklin MJ, Ford TE (2013). Bile salts affect expression of Escherichia coli O157:H7 genes for virulence and iron acquisition, and promote growth under iron limiting conditions. PLoS One.

